# “You can’t un-ring the bell”: a mixed methods approach to understanding veteran and family perspectives of recovery from military-related posttraumatic stress disorder

**DOI:** 10.1186/s12888-021-03622-3

**Published:** 2022-01-14

**Authors:** Kate St. Cyr, Jenny J. W. Liu, Heidi Cramm, Anthony Nazarov, Renee Hunt, Callista Forchuk, Erisa Deda, J. Don Richardson

**Affiliations:** 1 Dalla Lana School of Public Health, University of Toronto, Toronto, ON Canada; 2 Department of Psychiatry, Western University, London, ON Canada; 3 School of Rehabilitation Therapy, Queen’s University, Kingston, ON Canada; 4 Lawson Health Research Institute, London, ON Canada; 5 St. Joseph’s Operational Stress Injury Clinic, London, ON Canada

**Keywords:** Veterans, Posttraumatic stress disorder, Recovery, Mixed methods

## Abstract

**Background:**

Military-related posttraumatic stress disorder (PTSD) is a complex diagnosis with non-linear trajectories of coping and recovery. Current approaches to the evaluation of PTSD and treatment discontinuation often rely on biomedical models that dichotomize recovery based on symptom thresholds. This approach may not sufficiently capture the complex lived experiences of Veterans and their families. To explore conceptualizations of recovery, we sought perspectives from Veterans and their partners in a pilot study to understand: 1) how Veterans nearing completion of treatment for military-related PTSD and their partners view recovery; and 2) the experience of progressing through treatment towards recovery.

**Methods:**

We employed a concurrent mixed methods design. Nine Veterans nearing the end of their treatment at a specialized outpatient mental health clinic completed quantitative self-report tools assessing PTSD and depressive symptom severity, and an individual, semi-structured interview assessing views on their treatment and recovery processes. Veterans’ partners participated in a separate interview to capture views of their partners’ treatment and recovery processes. Descriptive analyses of self-report symptom severity data were interpreted alongside emergent themes arising from inductive content analysis of qualitative interviews.

**Results:**

While over half of Veterans were considered “recovered” based on quantitative assessments of symptoms, individual reflections of “recovery” were not always aligned with these quantitative assessments. A persistent narrative highlighted by participants was that recovery from military-related PTSD was not viewed as a binary outcome (i.e., recovered vs. not recovered); rather, recovery was seen as a dynamic, non-linear process. Key components of the recovery process identified by participants included a positive therapeutic relationship, social support networks, and a toolkit of adaptive strategies to address PTSD symptoms.

**Conclusions:**

For participants in our study, recovery was seen as the ability to navigate ongoing issues of symptom management, re-engagement with meaningful roles and social networks, and a readiness for discontinuing intensive, specialized mental health treatment. The findings of this study highlight important considerations in balancing the practical utility of symptom severity assessments with a better understanding of the treatment discontinuation-related needs of Veterans with military-related PTSD and their families, which align with a contemporary biopsychosocial approach to recovery.

**Supplementary Information:**

The online version contains supplementary material available at 10.1186/s12888-021-03622-3.

## Introduction

Posttraumatic stress disorder (PTSD) and other psychiatric conditions like depression, anxiety, and substance misuse resulting from military service [1] have been well-documented across numerous national militaries [2–8]. In Canada, the 2013 Life After Service Study, a population-based survey of Canadian Armed Forces (CAF) Veterans, found that 23.4% of respondents had a diagnosed mental health condition; of these individuals, 18% had probable PTSD [9]. PTSD is characterized by four symptom clusters: recurrent intrusive thoughts; avoidance of memories, thoughts, or external reminders of the traumatic event; negative alterations in cognition and mood; and increased reactivity [10]. Current best-practice guidelines for PTSD include a number of psychotherapeutic and pharmacologic interventions [11–16]. However, despite evidence that these treatments can lead to PTSD remission for many [17], studies of Veterans often report poorer treatment outcomes than their civilian counterparts [18] and may experience symptom exacerbation over time [19].

Although understandings of recovery have expanded beyond the dichotomization of presence/absence of symptoms, current practices often rely on tools largely developed out of biomedical frameworks, such as quantitative assessments of clinical symptoms. In this regard, individuals may be considered “recovered” if their symptom severity scores fall below a threshold [20]. Yet, quantifications of symptoms and function as assessed through various tools may differ from individual self-perceptions [21]. Indeed, evolving understandings of recovery can be defined as living with purpose and developing meaningful relationships with others in the community despite the absence or presence of symptoms [22, 23].

Despite this evidence supporting expanded views of recovery, much of the research examining mental health-related treatment outcomes amongst CAF members and Veterans has relied on dichotomized quantifications of recovery, which equates recovery with the abatement of clinical symptoms [24–26]. To date, there is little evidence available to describe how CAF Veterans personally define recovery from their service-related psychiatric conditions. Moreover, no study to date has explored relational understandings of recovery between Veterans and their close family members, despite evidence highlighting the meaningful roles family members play in contributing to both emotional and functional impacts of operational stress injuries (OSIs) [27–30].

This pilot study aims to fill existing gaps by seeking perspectives on recovery from service-related psychiatric conditions from Veterans and their partners. Specifically, the objectives of the study are to: 1) understand how CAF Veterans nearing completion of treatment for military-related PTSD and their partners conceptualize and understand recovery; and 2) explore the experiences of Veterans and their partners during through treatment.

## Methods

### Study design and recruitment

The current study employed a concurrent mixed methods design with quantitative evaluations of PTSD and depressive symptoms via psychometric tools and qualitative explorations of PTSD treatment and recovery through individual, semi-structured interviews under a pragmatic paradigm [31].

Data were collected from 1) CAF Veterans who have had engagement with a multidisciplinary clinical team for a military service-related mental health condition; and 2) their partners. Individuals were eligible to participate in the study if they: 1) were English-speaking CAF Veterans; 2) were receiving treatment for a service-related mental health condition from the St. Joseph’s OSI Clinic, a specialized, multidisciplinary clinic located in London, Ontario, Canada; 3) demonstrated clinically significant gains during treatment; 4) were between the ages of 18-65 years old at the time of recruitment; 5) were in a stable married or common-law relationship at the time of recruitment; 6) had a partner who was willing to participate in an individual interview about their experiences with their partner’s recovery; and 7) were able to provide informed consent.

A purposive sampling approach of Veterans nearing the completion of their treatment at the Clinic was employed. Psychiatrists providing care at the OSI Clinic were asked to identify Veteran clients who had demonstrated significant clinical gains and who were approaching remission from their PTSD symptoms, based on the professional judgement of their primary psychiatrist and as measured by the PTSD Checklist for DSM-5 (PCL-5) [32], a validated, self-report questionnaire that is administered at every treatment session. With the Veteran’s permission, their contact information was forwarded by the referring psychiatrist to the research team for eligibility screening and enrollment. Potential participants were contacted by a member of the research team by phone to discuss participation in the current study. Interested individuals were provided with information outlining the purpose and details of the study. After providing informed consent, interview sessions were scheduled. Participants were recruited into the study until thematic saturation [33] was reached, based on recurring discussions and consensus among the research team.

### Ethical considerations

Written informed consent was obtained from all participants. Although referrals to the study were made by the Veteran’s primary psychiatrist, the clinical team had no knowledge of who was ultimately enrolled in the study. Participants were free to withdraw consent at any time, and their decision to participate in the study had no impact on the access to the range of programs and services offered at the OSI Clinic. This study was approved by Western University’s Office of Human Research Ethics and the Lawson Health Research Institute.

### Data collection

Interviews lasting 90 min in duration were conducted at the OSI Clinic, or at a location requested by the Veteran and his or her partner (e.g., their home). Interviews with Veterans and partners were conducted simultaneously and separately in non-adjoining rooms to ensure confidentiality was respected. Interviews were conducted in an open style intended to capture participants’ lived experiences and personalized stories. Semi-structured interview scripts were developed to ensure that specific topics of interest to the present work were addressed. These included: the treatment process, social support, the role of the partner in treatment, and understanding of recovery. Examples of questions asked include, “How have your symptoms changed through your experiences at the OSI Clinic?” and “How has your significant other played a role in dealing with your PTSD?”. Standard prompts were used to elicit deeper nuance and granular detail regarding participant experiences (see Additional files 1 and 2 for interview guides).

### Measures

Directly following the interview, Veterans completed measures of military, clinical, and psychosocial factors. These included a demographic questionnaire, the PCL-5 [32], and the Patient Health Questionnaire-9 (PHQ-9) [34]. The PCL-5 is a twenty-item measure used to assess presence and severity of PTSD symptoms, across each of the DSM-5 PTSD symptom clusters. Respondents rate how frequently they have been bothered by each symptom over the past month using a five-point Likert-type scale, where 0 = “not at all” and 4 = “extremely”. Items can be summed for a total symptom severity score (range = 0 to 80); scores of 33 or higher are indicative of clinically significant symptoms of PTSD. A provisional PTSD diagnosis can also be made when a respondent endorses at least one cluster B item, one cluster C item, two cluster D items, and one cluster E item as “moderately” or higher [32]. The PHQ-9 is a nine-item measure used to assess presence and severity of depressive symptoms. Respondents rate how often they have been bothered by each of the DSM-IV depressive symptoms over the past two weeks using a four-point Likert-type scale, where 0 = “not at all” and 3 = “nearly every day”. Items are summed for a total score (range = 0 to 27). Total scores of 0-4, 5-9, 10-14, 15-19, and 20-27 are indicative of minimal, mild, moderate, moderately severe, and severe depression severity, respectively [34].

### Data analysis

Descriptive quantitative analyses were conducted using the Statistical Package for the Social Sciences (SPSS), version 26. Qualitative data from interviews were audio-recorded, transcribed verbatim, and analyzed thematically using NVivo 12 software [35]. Interviews were coded and analyzed using exploratory inductive content analysis [36].

All interviews were conducted by RH and AN. The first eight interviews (four dyads) were read and analyzed by HC, RH, CF, AN, and ED; initial codes were developed following an in-depth immersion in the content of interviews and discussion. The coding tree consisted of eleven main domains: recognizing something is wrong, pre-treatment coping, precipitating events, recovery is enduring, self-acceptance, cognitive reframing, behavioural management, defining recovery, recovery – partner, social support, and military culture. RH and CF performed an initial application of the coding tree on two interviews (one dyad) followed by an in-depth discussion to ensure consensus and calibration in subsequent application. The remaining interviews were coded either by KS, RH, or CF. Coded data from the initial eight interviews (four dyads) were extracted and analyzed to identify emerging themes. Seven themes were developed through discussion between HC, RH, AN, CF, and ED and refined by HC and RH. The final ten interviews (five dyads) were then coded by RH, CF, or KS; and HC further interpreted and refined themes. No new themes were identified at this point, and after discussion with HC, RH, and AN, the group reached consensus that thematic saturation had been achieved. Transcripts were not returned to participants for comment and/or correction.

## Results

### Sample characteristics

Participants were nine heterosexual dyads of CAF Veterans (mean age = 51.7, SD = 5.3) and their partners (mean age = 47.4; SD = 7.1). Since most treatment-seeking Veterans at the OSI Clinic are male (approximately 90%), our sample consisted exclusively of male Veterans and their female partners. At treatment intake, all nine Veterans had been diagnosed with military-related PTSD. Table 1, below, highlights the baseline characteristics of the study participants.

**Table 1:** Veterans’ sociodemographic, military, and clinical characteristics at study onset

Variable	Veteran (***N*** = 9)	Partner (***N*** = 7)
Sociodemographic & Military	Mean (SD)	Mean (SD)
Age (years)	51.7 (5.3)	47.4 (7.1)
Military service (years)	22.9 (7.9)	–
Number of deployments	4.9 (2.2)	–
	**N (%)**	
Highest level of education		
Completed high school	6 (66.7)	1 (14.3)
Some college	0 (0.0)	1 (14.3)
Completed college	1 (11.1)	5 (71.4)
Completed university	2 (22.2)	0 (0.0)
Income		
Missing	1 (11.1)	0 (0.0)
<$60,000	1 (11.1)	1 (14.3)
$60,000 to $99,999	0 (0.0)	2 (28.6)
$100,000 to $119,999	4 (44.4)	2 (28.6)
>$120,000	3 (33.3)	2 (28.6)
Lives with at least one child < 18 years of age	5 (55.6)	6 (85.7)
**Clinical**	**Mean (SD)**	
Time in treatment (years)	5.4 (2.2)	–
PCL-5 total	29.0 (14.1)	–
PHQ-9 total	7.6 (2.8)	–
	**N (%)**	
Meets screening criteria for PTSD (PCL-5 score of 33 or higher)	4 (44.4)	–
Meets PCL-5 provisional diagnostic criteria (1 cluster B, 1 cluster C, 2 cluster D, and 1 cluster E symptoms as “moderately” or higher)	4 (44.4)	–
Meets screening criteria for moderate or higher depressive symptom severity (PHQ-9 score of 10 or higher)	2 (22.2)	–

### Quantitative markers of recovery

Four Veterans scored at or above the standard cut-off value of 33 on the PCL-5 [32], indicating the presence of clinically significant symptoms of PTSD. The remaining five Veterans scored below the standard cut-off of 33. Four Veterans also met DSM-5 provisional criteria for a PTSD diagnosis, via the PCL-5. There was noticeable variability in the range of PCL-5 scores, however, with the lowest score being 2 and the highest being 51. We also assessed depressive symptom severity using the PHQ-9, as depression commonly co-occurs with PTSD. One Veteran scored in the “no depressive symptoms” range (range = 0 to 4), six scored in the “mild severity” range (range = 5 to 9), and two scored in the “moderate severity” range (range = 10 to 14) [37].

### Qualitative explorations of recovery

Seven themes regarding recovery emerged over the course of the analysis, organized chronologically in line with timeline of treatment as well as through shared lived experiences, including: 1) pre-treatment understanding of mental illness and coping; 2) motivations for seeking treatment; 3) recognizing the experience as PTSD; 4) the experience of treatment; 5) Veterans’ take-aways from treatment; 6) expanding understandings of recovery; and 7) the role of social support in mental health. We note that the role of social support is prevalent throughout the treatment and recovery processes, and is therefore the exception to the chronological ordering of the themes. Below, we highlight key components of each of the themes using salient quotes from study participants, whose real names have been replaced with pseudonyms.

I. *Pre-treatment understanding of mental illness and coping*:
  Participants expressed challenges related to awareness of their PTSD prior to engaging in treatment, behavioural disengagement, and sentiments of apathy and meaninglessness. One Veteran, Eric, stated, “*I didn’t know what the hell was going on, to be honest. I was just, I don’t know, adrift, didn’t know what the hell was going on.”* Another Veteran, Dean, found that he “*was next to oblivious to what was going on emotionally or personally or anything else, with my wife or kids or anything else.*” Eric’s and Dean’s pre-treatment experiences speak clearly to the lack of awareness of a greater psychological difficulty than simply re-adjusting to life as a civilian. Stephen described this period of his life as “chaos” and a time where he “*felt like my head was on fire, like literally like my brain was on fire.*”
  Partners also related Veterans’ challenges as associated with the transition from military to civilian life. For example, Michelle shared that her partner “…*went through this time where he was having severe anxiety attacks while he was still serving, and so we didn’t know what was going on. And I had taken him like in the middle of the night to the hospital because he felt like he was having a heart attack. We didn’t know anything about anxiety attacks, you know.*” Both the Veteran and partner in one dyad independently commented on the Veteran’s struggle with reintegration to civilian life. Julian likened the experience as “*always want[ing] to go back into the fire, I call it, right*?” His partner, Julia, described it as “*…it’s like they’re in another world. It’s like they just want to go back… they just want to go back.*”
  Participants described the coping strategies they adopted to manage some of the aforementioned experiences. Denial, hyperarousal, and avoidance were endorsed by the Veteran participants as ways of coping with or dealing with their PTSD symptoms. Eric describes his denial as: “…*kept my head down kind of thing and carried through life here. It was a freaking mess trying to work and deal with all these issues myself. But I guess I kind of buried it.*” Andrew similarly stated that “*for eighteen years, I sort of shoved it away, and tried not to dwell on it.*” Eric’s partner, Erika, also felt that Eric was denying that he was experiencing symptoms of PTSD. She stated, “*he was in denial even if I kept saying to him, you have PTSD.*” Veterans also spoke about using substances to help avoid dealing with their feelings. Julian reflected on his alcohol use, “*So dealing with my PTSD or my symptoms was dealing, you know, with the bottle. So. That’s why I drank so much, you know, to sleep, and I had to deal with what was going on in my head.”* Veterans’ partners also described the strategies they employed to deal with their partners’ symptom expressions. For example, Deanna stated, “…*it was still quite tough because it was still walking on eggshells [around him].*”

II. *Motivations for seeking treatment*:
  Veterans emphasized that interactions with military friends with lived experiences of PTSD, the urging of their partners, and noticing changes in their social networks acted as catalysts for seeking treatment. Eric shared, “*I think it was when I went to that reunion and met all my army buddies... And they just in talking, they were the ones I guess who kind of went, ‘Hey, you should really see someone.’ Because they were telling me their experience, I guess. They were like I don’t know, two or three, maybe four years into the process of healing.*” Dean recalled that when he finally started treatment, “*I was completely disconnected from everything, personal, socially.*” Veterans’ partners acknowledged the important role of relationships as motivation for treatment-seeking. For example, Deanna’s partner Dean agreed to seek treatment after she “…*told him he needed to go and get help or we were done. I said I wasn’t happy and I had noticed a big change since he had come home. I said he needed to go get help, or he needed to go.*” Julia reported that it was her partner’s close friend, who is also a military Veteran, who encouraged him to seek treatment: “*…he was, like, suicidal, and his friend, [name redacted], he reached out to him…and he got him in [to treatment].*”

III. *Recognizing the experience as PTSD*:
  Veterans reflected on how they came to the realization that they were indeed experiencing PTSD and the impact of that realization on their self-view. Given that militaries traditionally placed great value on masculinity and toughness, Veterans in this study reported mixed feelings of distress and relief. For example, Julian said, “*I cried like a baby, you know, for 15 minutes. I couldn’t believe that here I am, a [high ranking officer] and I’m broken*”. Julian credited psychoeducation about PTSD with shifting his perspective: “*So learning about mental health really turned my world around, turned my vision and, and said, hey, all these years I wasn’t crazy. You know. This is an injury.*” For many Veterans and partners, the official diagnosis served to provide a form of acknowledgment, justification, comfort, and relief. They also spoke about the realization of the impact of PTSD on the family unit, and about the accompanying guilt they felt. Julian said, “*You were injured, but look what you did you to your kids. Look what you did to your ex-wife… There’s only so much a person can take, right?*”
  Many partners remarked that they knew their partner was experiencing challenges prior to receiving a diagnosis, but they were often unable to identify precisely what was driving these issues. Michelle said, “*all I could do is help him deal with the symptoms. Because I didn’t know kind of what was going on either, I didn’t understand it*.” Andrea acknowledged that she didn’t identify her partner’s symptoms as PTSD until “…*after the fact…*”, when they “*…just overpowered him…*”.

IV. *The experience of treatment*:
  Veterans described their treatment process as non-linear, at times challenging, and often lengthy. Nathan stated that, “*There were times that I was like, man, is this ever going to end?”* Luke said that his experience with the treatment process was “*trial and error, see if this works, see if this medication helped you out.*” Nathan also spoke of frustration early in the treatment process. He shared that he felt he had little control over his progress, stating, “*…you could progress good, you could progress bad, you don’t have control of anything really.*” Stephen echoed feeling frustrated early in the process, primarily due to challenges finding a clinician who provided a good fit for his needs. Once that hurdle had been overcome, he indicated that he made significant treatment gains: “*[clinician 1] and [inpatient facility 1]…are the two most important things that have happened to me to help me recover to where I am now.*” Several Veterans remarked upon the importance of actively engaging in their treatment. Eric stated that he learned, “*You have to put the effort into recovery, healing yourself…if you don’t put the effort into it, you’re going to get no result at all.*” Julian echoed this sentiment: “*You only get what you put into it, right?*”
  Many partners reported taking an active role in the Veterans’ treatment. Michelle shared that she “*went to every appointment with him,*” and Deanna added that she participated in treatment sessions with Dean so that she “*…would have a better understanding so that I could support him*.” Julia found attending sessions particularly beneficial because it facilitated “*implement[ing] [strategies learned in therapy] at home and it’s like wow.*”

Partners also reported an increased understanding of what PTSD is and how it manifests through attendance at the Veterans’ treatment sessions, as well as a more thorough understanding of the specific experiences and traumatic events the Veterans had been working through. Deanna shared that attending treatment sessions with her partner “*…was helpful to give me a better understanding of what he was going through […] a better understanding of why he sometimes be the way that he was.*”

V. *Veterans’ take-aways from treatment*:
  Participants also spoke candidly about how they perceived the outcomes of their treatment. For example, Andrew said, “*I don’t know if I’m better, I don’t know if I’ll ever be. But I’m definitely better than I was when I was in the dark time.*” Luke described a similar sentiment, stating that while he feels “*more able to manage,*” but that, in terms of being symptom-free, “*…I won’t get there*. *.. it’s been 23 years, and I still don’t feel that I’m at an acceptable level.*” Nathan reported a contrasting experience, stating that, pre-treatment, “*I certainly didn’t think that I was going to feel normal again*” but reports that, following treatment, “*I just feel good.*” Increased self-awareness was consistently identified as being integral to effectively employing these strategies. For example, Dean stated, “*I can now recognize, sort of, the internal physical changes that come with the anxiety rising […] and I can feel that happening, and I can identify it.*” This increased self-awareness allowed Veterans to recognize how they felt and what tool they needed to employ to address their current need instead of engaging in their pre-treatment maladaptive coping strategies.

Veterans also spoke about learning how to create conditions that would set them up for success in navigating stressful external stimuli. Dean stated, “*…I can’t go back to just being wherever, doing whatever and adapting to the situation as it goes. I need to sort of prepare and plan more for it than I used to.*” The ability to plan for success, for many Veterans, included striking an adaptive balance between avoidance and engagement. Eric described a situation in which he carefully planned out the steps he needed to take to enjoy a crowded event that he wanted to attend but could have been quite stressful without adequate planning. Michael spoke of a similar situation that left him overwhelmed, exhausted, and not keen to repeat until he had a more appropriate strategy in his toolbox. This balance between avoidance and engagement, combined with careful planning, allowed Veterans and their partners to participate in social activities, such as travelling with friends and attending concerts. Veterans reported developing affective, behavioural, and cognitive strategies to assist in their symptom management. Eric commented on the utility of having a “toolbox” of strategies to select from when he found himself experiencing symptoms: “*You learn how to ground, that’s a tool. You learn how to calm yourself, that’s another tool. And then being able to pick the right tool appropriately.*”

VI. *Expanding understandings of recovery*:
  Veterans and their partners reported mixed views of what recovery meant to them. Dean shared that he entered treatment with the perception that he would “*go back to normal,*” while Julian stated that recovery from PTSD is “*unobtainable*” and that “*nobody will ever be 100 percent recovered.*” Michael similarly reported that, with PTSD, “*you can’t un-ring the bell, once you’ve seen something or smelled it or heard it it’s always with you*”, emphasizing his perspective that the consequences of military-related PTSD are profound and long-lasting.
  Many debated the relationship of symptoms to recovery. For example, Dean felt that “*the concept of recovery is…it’s easy to define, with regards to scores on questionnaires, because basically you’re defining people as not clinically acute at this time.*” Veterans also indicated that recovery from PTSD was incompatible with a binary conceptualization of recovered vs. non-recovered. Partners of Veterans echoed this sentiment. Erika likened recovery from PTSD to a chronic medical illness: “*I know that PTSD is not a thing that’s going to go away. So it is the same thing as diabetes. You’re a diabetic, you’re going to stay a diabetic, and it’s the same with PTSD.*” John commented that he generally feels himself to be recovered, but feels as though his partner believes that he’s “*recovered now to a point but not fully,*” because “*fully would be probably somebody who doesn’t really have any real issues.*” This demonstrates some discordance between Veterans’ and their partners’ perceptions of recovery.

Veterans spoke of how their perceptions of recovery changed over time, and how they had to rethink the outcomes that engagement in treatment would allow. Dean commented, “*I can understand, now, that I’ll never go back to doing the same things in the same ways that I did.*” Michelle agreed with this sentiment, stating that “*you learn to live with things, you know, like you learn to do things he* can *do*.” Julia remarked on how she perceived recovery as a temporary state: “*…when you say recovery, I feel like it’s permanent – it’s like a permanent state of well-being. I don’t see that. It is temporary, that he’ll last for a very long time, but that does not guarantee the slip-back.”* This sentiment was echoed by some of the Veterans. Eric shared, “*If I’m having a good day, then that’s fine, then I feel recovered. But if I’m having a bad day, then I don’t feel so recovered.*” Ongoing “maintenance” therapy – such as periodic check-ins with a healthcare professional – was also viewed by many participants to be a key element in remaining in a state of recovery.

VII. *The role of social support in mental health*:
  Shrinking social networks were commonly described by the Veterans and partners in our sample. Some respondents attributed this partly to the perceived stigma around PTSD. Eric said, “*This is just stuff […] that you didn’t talk about.*” Veterans reported that when they did open up to other Veterans about their experiences, they arrived at the vital realization that they were “*not the only one*” (Eric) going through these challenges. For some Veterans, there was a distinct withdrawal from larger social networks and a reprioritization of the relationships that were most important to them throughout the treatment process. Many Veterans appeared to feel at ease with their new social norms. Dean shared, “*So I’m not sort of engaged very much in a larger circle of people…social role is much smaller, but’s also stabilized.*” Partners also commented on changes within their spousal relationships and within their social networks. Andrea shared that, “…*we’re not just existing now, so that’s a bonus. And you know, he’s starting to talk to some of our friends more, which is good, because he wasn’t talking to them either…but he’s doing better, he’s doing better with more people*.”

Support from military peers was highlighted as an integral part of Veterans’ treatment and recovery. Julian highlighted the importance of social programs offered through the Canadian Legion and other similar organizations that aimed to maintain some semblance of having a military family, which was something he felt deep loss about when he left the military. He stated these programs provided a safe space for Veterans to “*get out and socialize*”, and reduce isolation by getting them “*out there to the community…meeting people.*” Julian’s wife, Julia, also commented on the significant role played by these military-specific social programs. She reported that “*[h]e loved being there for all the other soldiers*”, and that it was obvious he was passionate about providing the same support he had appreciated so much.

Finally, Veterans also commented on the protective role the support of their partners played in their recoveries. Andrew shared that his partner has been “*his rock*” throughout the treatment process, and with her support, “*I smile more than I ever have. And the light of my day is just having [Andrea] smile.*” John shared that his partner, Johanna, was “*always there like no matter what sort of condition I was feeling or in.*” Partners also reflected on the role they played in their partners’ recoveries. Michelle acknowledged that she was heavily involved throughout her partner’s treatment, stating that, “*I think he would give up if I wasn’t around.”* Julia also commented on the importance of supporting her partner through treatment, saying her role was “*…more of being there and being supportive because he always just tells me that I just need you to be here…I just feel like he just needed somebody to stand by him*.”

### Integration of quantitative and qualitative findings

To integrate quantitative and qualitative findings, we merged each Veterans’ quantitative data with their own perceptions about whether they considered themselves recovered. Interestingly, symptom severity scores were, at times, discordant with participants’ narratives of recovery. Some individuals with higher levels of symptom severity reported a less positive outlook related to their own recovery. For example, Luke, whose PTSD and MDD symptom severity scores remain clinically significant, indicated that recovery is “*tough, it’s draining, it’s tiring, it’s exhausting, frustrating…it never becomes easier.”*. Other participants with high levels of symptom severity indicated that the results of the questionnaires are not truly reflective of how they feel – that, despite their high symptom severity scores, they feel well on the path to recovery. For example, Stephen said that, “*I do those questionnaires and I feel like I’ve improved and then I talk to [my clinician] and he’s like no, your scores are here and here…*”. Conversely, participants with lower levels of symptom severity, indicative of recovery, did not necessarily report narratives consistent with their questionnaire data. Andrew, who no longer met the PCL-5 cut-off for probable PTSD and whose PHQ-9 score fell in the mild range, said, “*I don’t know if I can honestly say I’m better…I know I’m not like I used to be, so I know I’m not 100%.”*

Beyond evaluation of symptoms in PTSD, an overarching theme emerging from both Veterans and their partners’ accounts of experiences with treatment is their evolving understanding of what it means to live with PTSD. In this sample, recovery from military-related PTSD was seen as much more complex than the dichotomous presence/absence of symptoms. Instead, recovery included acknowledging the ongoing presence of milder or fluctuating symptoms and having the toolkit to manage them (e.g., Erika’s comment “*I know that PTSD is not a thing that’s going to go away.”*). It also involved finding new ways to engage and live meaningfully within their networks and to establish purposeful connections and re-engage with living their lives, despite the reality that there may be a level of symptoms necessitating ongoing self management. For example, Michael stated that “*You know, it’s – living is about a life well-lived…that’s what matters.”* Partners also reported being able to re-engage with aspects of their lives that had become neglected. Andrea shared, “*I’m happier now myself too, because I know that…he can smile again, so I know he’s okay to a certain extent.*” Finally, recovery was viewed as moving towards a readiness to discontinue intensive treatment and, for some, shifting to less frequent “maintenance” therapy. For example, Julia shared that, while she agreed her partner’s functioning had improved over the course of treatment, she didn’t believe her partner would ever be able to stop taking medication for his symptoms because *“if he did he would be, like, right back down…otherwise he just - flashbacks*”. Nathan reported feeling at peace with his current lower-intensity treatment regime, but that he “*keep[s] in the back of my mind that there’s a possibility of falling back into – I call it falling off the rails and having a relapse*.”

## Discussion

The current paper explored understandings of recovery from veterans and their partners using a mixed methods approach. Using a quantitative evaluation of recovery, nearly half of the Veterans were considered “recovered” via use of the recommended PCL-5 scoring cut-off. Qualitatively, narratives of recovery illustrate a non-linear approach of understanding and living with PTSD. Importantly, understandings of recovery across qualitative and quantitative means were not always consistent with one another, suggestive of the complexities and differences in the interpretation of what “recovery” means for Veterans living with PTSD and their family members. Indeed, prior research has documented the complexities and non-linearity of recovery from PTSD, and highlighted the important roles of fear and stigma, personal beliefs (e.g., upholding military ideals such as strength), supportive networks and family members, sense of belonging, and the resilience of living with a mental illness in contributing to processes of recovery [38–40]. For those with PTSD, their experiences and goals of treatment may extend beyond termination of specific symptoms (whether PTSD or comorbidities). Treatment-seeking Veterans may not focus their goals on symptoms, and instead, focus on how they are able to function and resume activities and social relationships. Together, findings from the current paper add to a growing body of literature that underscores the dynamic and interactive process of recovery as extending beyond a symptom abatement or returning to the person they were prior to their military-related traumatic experiences.

In the current sample, participants’ recovery experiences were often markedly different from the dichotomization of illness and health from a biomedical perspective. Veterans and their partners reflected on the process of recovery as dynamic versus an end-state. Some felt that, once intensive therapy was no longer deemed necessary, maintaining an ongoing therapeutic relationship on a less frequent or formal basis was imperative to preserving the gains made in treatment. This perceived ongoing need for “maintenance” therapy parallels experiences with other chronic illnesses, such as diabetes. Future research exploring treatment outcomes of maintenance therapy may help assess the clinical and cost-effectiveness of such an approach, as well as potential implications on health programming and policy. Regardless, once a degree of stability is achieved, it is important to revisit the need for intense clinical interventions versus moving towards a more autonomous disease-management approach. In practice, mental health professionals often rely on symptom reduction thresholds as measured by clinician-administered and self-report questionnaires to guide treatment decisions. While the practical utility of these tools are important considerations in practice, Veterans receiving treatment for mental health conditions, such as PTSD, may benefit most when decisions around treatment termination are made using standard measures of symptom severity combined with the Veteran’s perceived readiness to navigate symptom exacerbation with the appropriate tool from his or her kit, as well as ability to function and engage meaningfully within their networks. Partners can provide invaluable contextual information and should be included in this determination whenever possible.

Findings from the current sample also emphasized distinctions between recovery and a readiness to discontinue treatment. Veterans and their partners expressed that being symptom-free for a prolonged period of time was unobtainable, but that being “recovered” could instead mean having the self-awareness and tools needed to manage symptoms of PTSD when they did appear. This finding echoes the work of Anthony [22] and Deegan [23], who suggested that recovery from mental illness does not necessarily equate to a lack of symptoms, but rather a life lived meaningfully in spite of them. At a conceptual level, these narratives reflect that readiness to discontinue treatment may be a separate concept not defined by the quantification of symptoms. Instead, the uncoupling from treatment dependence may be rooted in other factors, such as the ability to manage their symptoms and re-engage meaningfully in their lives.

Finally, findings highlight the importance of social identity and functioning as both motivations to seek treatment as well as readiness to discontinue treatment. Veterans identified social interactions as opportunities to reflect on their functioning. Further, partners’ engagement and support were often instrumental in Veterans’ treatment-seeking process. Indeed, research has suggested that mental illness is not an individual diagnosis, but rather experienced collectively in families and social networks [41, 42]. Taken together, considerations should be made to involve families in the treatment process when practical or appropriate, and to align the goals of treatment to support meaningful re-engagement and functioning in social networks.

The contributions of this study are marked by several strengths and limitations. First, the use of a mixed-methods approach allowed us to compare typical measures of treatment success and recovery with Veterans’ own perceptions of what it means to be recovered from military-related PTSD. Second, the inclusion of Veterans’ partners helps capture additional aspects of the treatment and recovery process that the Veteran may have overlooked, due to the intensity of military-related PTSD and the treatment process. It also provides further insight into how military-related PTSD impacts families and the scope of that impact. Our study was limited by the use of convenience sampling and only one type of dyad: male Veterans in stable relationships with female partners. In addition, the heterosexual dyads may have limited our scope of recovery from heteronormative perspectives as recovery may be defined differently by female Veterans, Veterans not in romantic or monogamous relationships, or Veterans in non-heterosexual relationships. The Veterans in this study reported multiple deployments, tended to be middle-aged, with higher average income levels, and were recruited from a single, specialized outpatient mental health clinic for members and Veterans of the CAF. It is possible that our findings are not generalizable to other Veteran populations. Finally, the current study explored quantitative and qualitative concepts of recovery. While clinical assessment tools are often used to make determinations regarding patients’ progress towards recovery, we recognize that clinical practices within and outside of Veteran communities may engage multiple sources of information in clinical decision-making processes.

## Conclusion and future directions

This study provides valuable information about the treatment and recovery processes for mental health clinicians who work with treatment-seeking Veterans. Findings shed additional light upon the relationship between symptom reduction and perceived ability to self-manage symptoms of PTSD and, perhaps more importantly, live meaningfully. The lack of congruency between standardized tools assessing recovery and self-perceptions of recovery highlight potential limitations in the use and reliance of dichotomous model of recovery that is based primarily on symptom screening tools. The use of these tools independent of consultations with Veterans and their families may affect treatment orientation and motivations. Meanwhile, the engagement of Veterans in treatment planning decisions along with their family members offers new opportunities that may affect the acceptability and understanding of mental illness diagnoses and recovery trajectories. Future research building upon these findings will help move us closer towards a working definition of recovery from military-related PTSD and alleviate some of the burden placed on Veterans and their families as they navigate the multitude of challenges associated with military-related PTSD.

## Supplementary Information

**Additional file 1.** Interview guide – Veterans.

**Additional file 2.** Interview guide – partners.

## Data Availability

The datasets generated and/or analysed during the current study are not publicly available due being part of an ongoing program of research, but de-identified data are available from the corresponding author on reasonable request.
