# Supplementary material for: “You can’t un-ring the bell”: a mixed methods approach to understanding veteran and family perspectives of recovery from military-related posttraumatic stress disorder
Source: BMC Psychiatry. 2022 Jan 14;22:37. doi: 10.1186/s12888-021-03622-3 (PMC8759247; doi:10.1186/s12888-021-03622-3)
Supplement: Supplementary file 1 — Additional file 1. Interview guide – Veterans. [file 12888_2021_3622_MOESM1_ESM.docx]

**Additional file 1**: Interview guide – Veterans

Interview number: ____________________

TURN ON TAPE RECORDER

**Section 1**: EXPERIENCES WITH THE TREATMENT PROVIDED AT OSI CLINIC

1. How have your symptoms changed through your experiences at the OSI Clinic?

Examples of probing questions:

1) What has your experience with the treatment provided been like?

a. How has this experience changed over the course of treatment?

2) How would you describe your life prior to receiving treatment?

3) How would you describe your life since beginning treatment and as you have moved through the process?

**Section 2**: DEFINING THE ROLE OF THE SIGNIFICANT OTHER DURING TREATMENT

1. How has your significant other played a role in dealing with your PTSD?

Examples of probing questions:

1) How would you describe your relationship with your significant other before entering treatment at the OSI Clinic?

2) How would you describe your relationship with your significant other since beginning treatment and as you’ve moved through the treatment process?

3) Tell me about the support you received from your significant other.

**Section 3**: DEFINING RECOVERY

1. What is it like to be recovering from PTSD?

Examples of Probing questions:

1) Would you consider yourself recovered? Please explain.

2) What factor(s) do you believe contributed the most towards your recovery?

3) What kind of changes have you noticed in yourself?

4) What differences do you think others might have recognized?

That concludes the interview. Do you have any other thoughts on what we have discussed today that you would like to share?
