# Supplementary material for: “You can’t un-ring the bell”: a mixed methods approach to understanding veteran and family perspectives of recovery from military-related posttraumatic stress disorder
Source: BMC Psychiatry. 2022 Jan 14;22:37. doi: 10.1186/s12888-021-03622-3 (PMC8759247; doi:10.1186/s12888-021-03622-3)
Supplement: Supplementary file 2 — Additional file 2. Interview guide – partners. [file 12888_2021_3622_MOESM2_ESM.docx]

**Additional file 2**: Interview guide – partners

Interview number: ____________________

TURN ON TAPE RECORDER

**Section 1**: EXPERIENCES WITH THE TREATMENT PROVIDED AT OSI CLINIC

1. How have your significant other’s symptoms changed through their experiences at the OSI Clinic?

Examples of probing questions:

1) From your perspective, what has their experience with the treatment provided been like?

a. How has this experience changed over the course of treatment?

2) How would you describe their life prior to receiving treatment?

3) How would you describe their life since beginning treatment and as they have moved through the process?

**Section 2**: DEFINING THE ROLE OF THE SIGNIFICANT OTHER DURING TREATMENT

1. How have you played a role in dealing with your significant other’s PTSD?

Examples of probing questions:

1) How would you describe your relationship with your significant other before they entered treatment?

2) How would you describe your relationship with your significant other since they began treatment and as they have moved through the treatment process?

3) Tell me about the support you have provided to your significant other.

**Section 3**: DEFINING RECOVERY

1. What is it like for your significant other to be recovering from PTSD?

Examples of probing questions:

1) Would you consider your significant other recovered? Please explain.

2) What factor(s) do you believe contributed the most towards your significant other’s recovery?

3) What kind of changes have you noticed in your significant other?

4) What differences do you think others might have recognized?

That concludes the interview. Do you have any other thoughts on what we have discussed today that you would like to share?
